# Dexamethasone Treatment Increases the Intracellular Calcium Level Through *TRPV6* in A549 Cells

**DOI:** 10.3390/ijms21031050

**Published:** 2020-02-05

**Authors:** Bo-Hui Jeon, Yeong-Min Yoo, Eui-Man Jung, Eui-Bae Jeung

**Affiliations:** Laboratory of Veterinary Biochemistry and Molecular Biology, College of Veterinary Medicine, Chungbuk National University, Cheongju, Chungbuk 28644, Korea; hui0416@naver.com (B.-H.J.); yyeongm@hanmail.net (Y.-M.Y.); jemman@hanmail.net (E.-M.J.)

**Keywords:** calcium, dexamethasone, TRPV6, A549 cells

## Abstract

This study investigated the effect of dexamethasone (DEX) on intracellular calcium levels and the expressions of transient receptor potential cation channel subcomponent V member 6 (*TRPV6*), sodium-calcium exchanger 1 (*NCX1*), and plasma membrane calcium ATPase 1 (*PMCA1*) in A549 cells. The intracellular calcium level, by using the calcium indicator pGP-CMV-GCaMP6f, increased following DEX treatment for 6, 12, and 24 h in A549 cells. In addition, Rhod-4 assay after DEX treatment for 24 h showed that DEX increased the level of intracellular calcium. The expression of the calcium influx *TRPV6* gene significantly increased, whereas the expressions of the calcium outflow *NCX1* and *PMCA1* genes significantly decreased with DEX treatment. The mRNA levels of surfactant protein genes *SFTPA1*, *SFTPB*, *SFTPC*, and *SFTPD* and the secreted airway mucin genes *MUC1* and *MUC5AC* were investigated by treating cells with DEX. The DEX treatment decreased the mRNA levels of *SFTPA1* and *SFTPB* but increased the mRNA levels of *SFTPC* and *SFTPD*. The *MUC1* mRNA level was increased by DEX treatment, whereas *MUC5AC* mRNA was significantly decreased. These results indicate that DEX influences the intracellular calcium level through *TRPV6*, and affects pulmonary surfactant genes and secreted airway mucin genes in A549 cells.

## 1. Introduction

Calcium is known to regulate a variety of cellular processes by activating or inhibiting cell signaling pathways or calcium regulatory proteins [1,2]. In the lungs, calcium has an important role in the maintenance of the extracellular structure, the function of surfactant materials, and the regulation of mucus secretion [3].

The transient receptor potential cation channel subfamily V member 6 (TRPV6), a representative calcium influx channel, is a channel associated with the progression of various cancers including prostate and breast cancers. The expression of TRPV6 is reported to be associated with tumor progression [4,5]. Calcium efflux through the plasma membrane is primarily accomplished by primary active transport via the sodium-calcium exchanger (NCX) and plasma membrane calcium-ATPases (PMCA). Overexpression of PMCA in HeLa cells is known to increase resistance to apoptosis. PMCA has been actively studied in most cancer cells, but studies on NCX have not been reported [2].

Pulmonary surfactants are protein–lipid complexes that cover and protect the surface of the alveoli and prevent their collapse. Pulmonary surfactants are composed of four proteins (*SFTPA1*, *SFTPB*, *SFTPC*, and *SFTPD*) that are produced by type II alveolar epithelial cells such as A549 [6]. Surfactant proteins are reported to be involved in the regulation of the immune system, and in enhancing the absorption of surfactant phospholipids into the air–liquid interface and reducing the surface tension within the lungs [7,8]. Pulmonary surfactant level is widely used as a biomarker of lung cancer [9]. An increase in cytoplasmic calcium influences the secretion of surfactant proteins [10].

Mucins are expressed in the epithelial cells of various organs and have a role in preventing or protecting against lung damage. They are also known to have an important role in the development of the lung. Mucins are divided into two types: membrane-bound mucus such as MUC1 and secretory mucus such as MUC5AC [11,12]. Corticosteroids can affect mucus expression as a part of the anti-inflammatory response and changes in the expression of calcium are associated with mucin secretion changes [13,14].

Recently, calmodulin-based gene-coding fluorescent calcium indicators (GCaMP-s) and genetically encoded Ca^2+^-indicators for optical imaging (GECO) have been used in many studies as they are a powerful tool for use in calcium imaging. The GCaMP6f indicator used in this study induces the formation of a complex with the calcium target peptide binding to calmodulin, which allows the detection of calcium signals by using GCaMP6f [15,16]. The GECO1.2 indicator can efficiently monitor changes in Ca^2+^ concentrations in cultured mammalian cells and is a promising technique for real-time cell activity research [17]. The Rhod-4 assay is a fluorescence-based assay for detecting intracellular calcium mobilization. Rhod-4, the brightest red of the calcium indicators, is an ideal indicator for the measurement of cellular calcium and, compared to Fluo-8, Rhod-4 is more photostable, making Rhod-4 based fluorescence imaging more robust [17].

Glucocorticoids are steroid hormones that are involved in various cellular and molecular systems and are used in antitumor chemotherapy. Synthetic glucocorticoids, such as dexamethasone (DEX), are one of the most commonly prescribed anti-inflammatory drug types and have been shown to have anticancer efficacy in tumor cells, such as those in prostate and lung cancers; moreover, glucocorticoids are also involved in calcium metabolism and absorption [11,18]. Since there is a lack of DEX research related to calcium regulation in A549 cells, this study was undertaken to investigate the effect of DEX on intracellular calcium levels and on the expressions of the *TRPV6*, *NCX1*, and *PMCA1* genes.

## 2. Results

**Figure 1:**
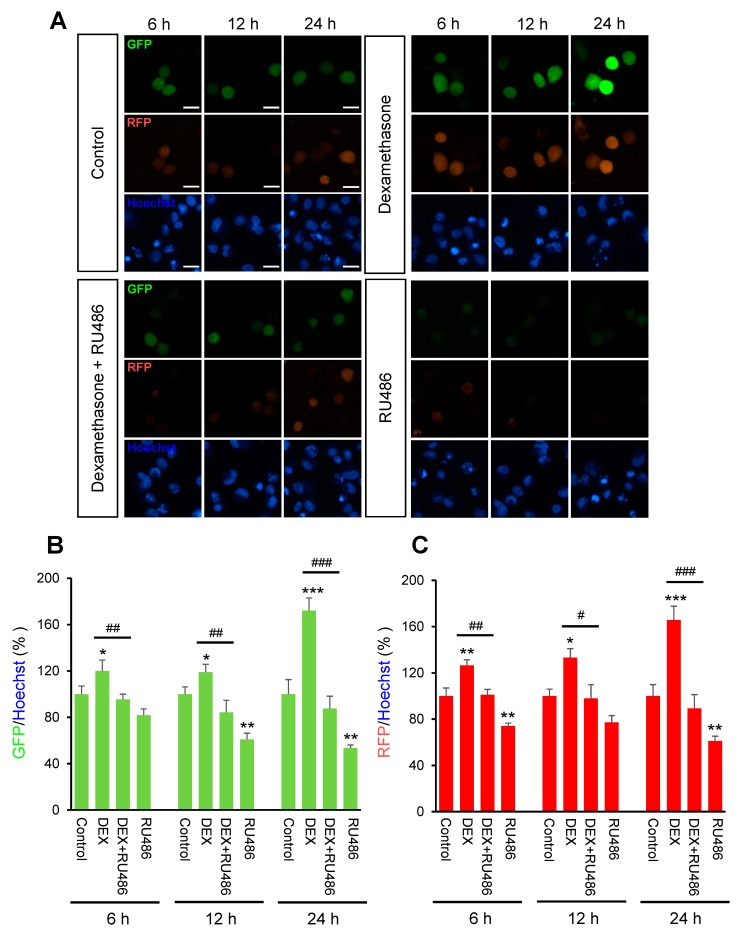
The intracellular calcium levels under dexamethasone treatment for 6, 12, and 24 h. A549 cells were seeded at 3 × 10^5^ in coverglass-bottom dish for microscopy and co-transfected with 0.5 μg of pGP-CMV-GCaMP6f and CMV-R-GECO1.2 then 1.5 μL of Lipofectamine in 50 μL of Opti-MEM medium at room temperature for 5 min. Intracellular calcium levels were increased by dexamethasone (DEX) treatment for 6, 12, and 24 h after pGP-CMV-GCaMP6f and CMV-R-GECO1.2 transfection. Increased intracellular calcium levels by DEX were determined by using lionheart microscopy. (**A**) Expression of pGP-CMV-GCaMP6f (green) and CMV-R-GECO1.2 (red) detected after 6, 12, and 24 h co-transfection. Nuclei were stained with Hoechst (blue). (**B**) The green fluorescent protein (GFP) intensities by pGP-CMV-GCaMP6f (green) were plotted for each of the 6, 12, and 24 h groups. (**C**) The red fluorescent protein (RFP) intensities by CMV-R-GECO1.2 (red) were plotted for each of the 6, 12, and 24 h groups. * *p <* 0.05, ** *p <* 0.01, *** *p <* 0.001 versus control; ^#^ *p <* 0.05, ^##^ *p <* 0.01, ^###^ *p <* 0.001 versus DEX. Scale bar, 25 μm.

**Figure 2:**
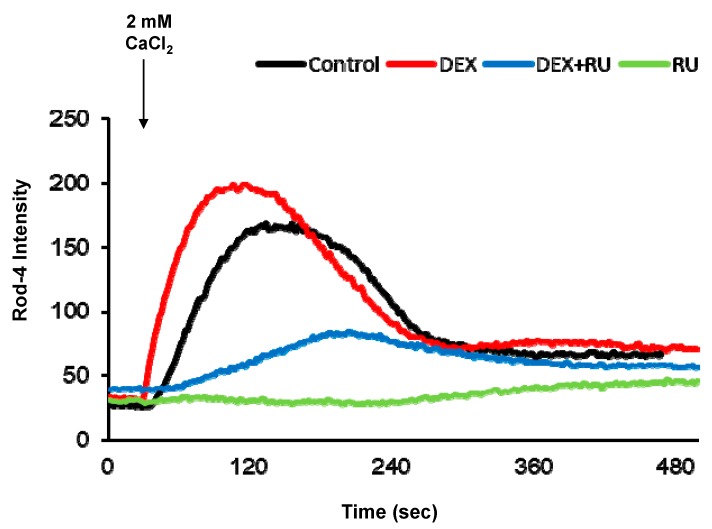
Intracellular calcium response affected by dexamethasone. Intracellular calcium response was increased by DEX at 24 h after attachment. DEX and a glucocorticoid receptor antagonist RU486 affected intracellular calcium response. Comparison of intracellular calcium response determined by confocal microscopy. DEX, 10^−8^ M of dexamethasone; DEX + RU, 10^−8^ M of dexamethasone treated with 10^−6^ M of RU486; RU, 10^−6^ M of RU486.

Initially, we investigated the effect of DEX on intracellular calcium levels. By using genetically encoded fluorescent calcium indicators, pGP-CMV-GCaMP6f (green, Figure 1A,B) and CMV-R-GECO1.2 (Red, Figure 1A,C), the intracellular calcium concentration in A549 cells was shown to be significantly increased by DEX treatment for 6, 12, or 24 h, whereas a glucocorticoid receptor antagonist RU486 treatment reduced the DEX treatment effect. After DEX treatment for 24 h, intracellular calcium mobilization was detected by using Rhod-4 assay (Figure 2). DEX significantly increased intracellular calcium concentration while RU486 treatment reduced the increase in DEX-induced calcium concentration. The treatment of RU486 alone abolished the increase in intracellular calcium.

**Figure 3:**
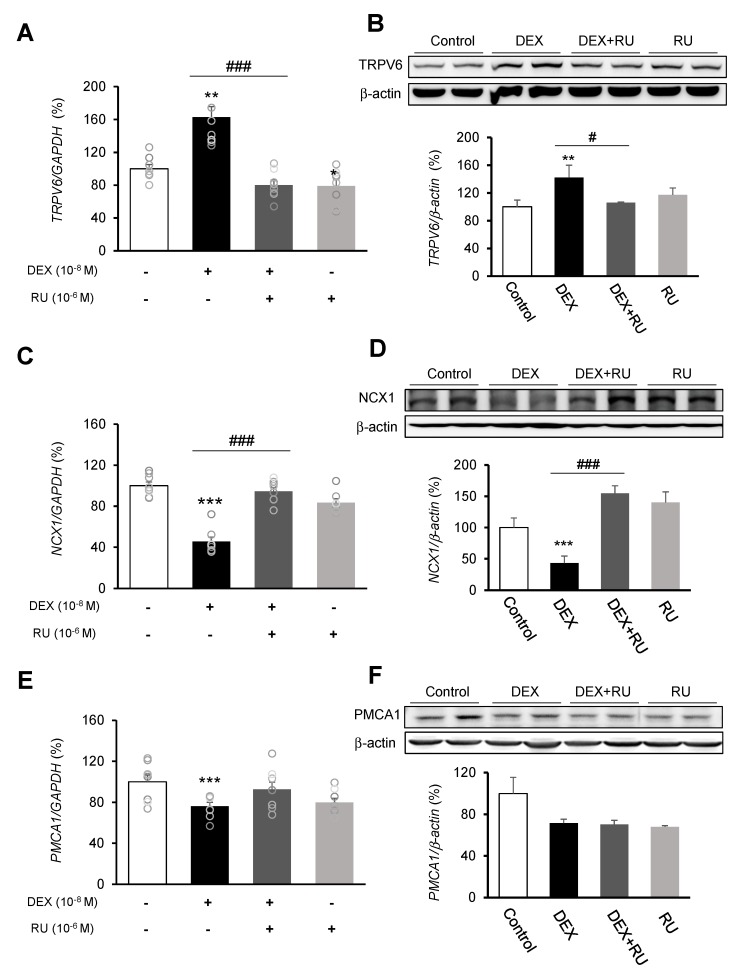
Regulation of calcium-processing gene expression by dexamethasone in A549 cells. Effect of DEX and its antagonist (RU486) on a transcriptional level of (**A**) transient receptor potential cation channel subfamily V member 6 (TRPV6) by real-time PCR, (**B**) TRPV6 by Western blotting, (**C**) sodium-calcium exchanger (NCX1) by real-time PCR, (**D**) NCX1 by Western blotting, (**E**) plasma membrane calcium ATPase 1 (PMCA1) by real-time PCR, and (**F**) PMCA1 by Western blotting. The mRNA level was measured by performing real-time PCR and was normalized by GAPDH. Quantification of protein levels determined by using NIH ImageJ software. Protein level was normalized by β-actin. * *p <* 0.05 versus Control; ** *p <* 0.01 versus Control; *** *p <* 0.001 versus control; ^#^ *p <* 0.05 versus DEX; ^#^^#^ *p <* 0.01 versus DEX; ^###^ *p <* 0.001 versus DEX.

**Figure 4:**
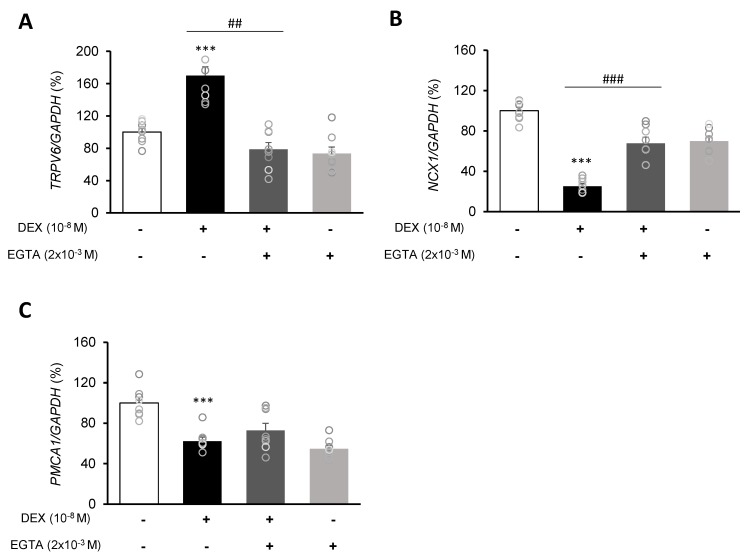
Effect of EGTA on calcium-processing genes in A549 cells. Effect of EGTA and DEX on the transcriptional level of (**A**) transient receptor potential cation channel subfamily V member 6 (TRPV6), (**B**) sodium-calcium exchanger (NCX1), and (**C**) plasma membrane calcium ATPase 1 (PMCA1) by real-time PCR. The mRNA level was measured by performing real-time PCR and was normalized by GAPDH. *** *p <* 0.001 versus control; ^##^ *p <* 0.01 versus EGTA; ^###^ *p <* 0.001 versus EGTA.

Following DEX treatment of A549 cells for 24 h, expressions of the calcium-processing genes *TRPV6*, *NCX1*, and *PMCA1* were examined. In addition, to determine whether the intracellular calcium concentration is affected by DEX, the mRNA levels of the *TRPV6*, *NCX1*, and *PMCA1* calcium-processing genes were examined following treatment with the calcium-specific chelating agent EGTA. Expression of *TRPV6* was significantly increased in the DEX-treated group compared to the control group, whereas the increase in the *TRPV6* level was reversed by using the DEX antagonist RU486 or the calcium chelator EGTA (Figure 3A,B and Figure 4A). Expressions of *NCX1* and *PMCA1* were significantly reduced in the DEX-treated group, and those increases were reversed by DEX plus RU486 or EGTA treatment (Figure 3C–F and Figure 4B,C). These results suggest that DEX regulates *TRPV6*, *NCX1*, and *PMCA1* expressions and produces an increase in intracellular calcium concentration.

**Figure 5:**
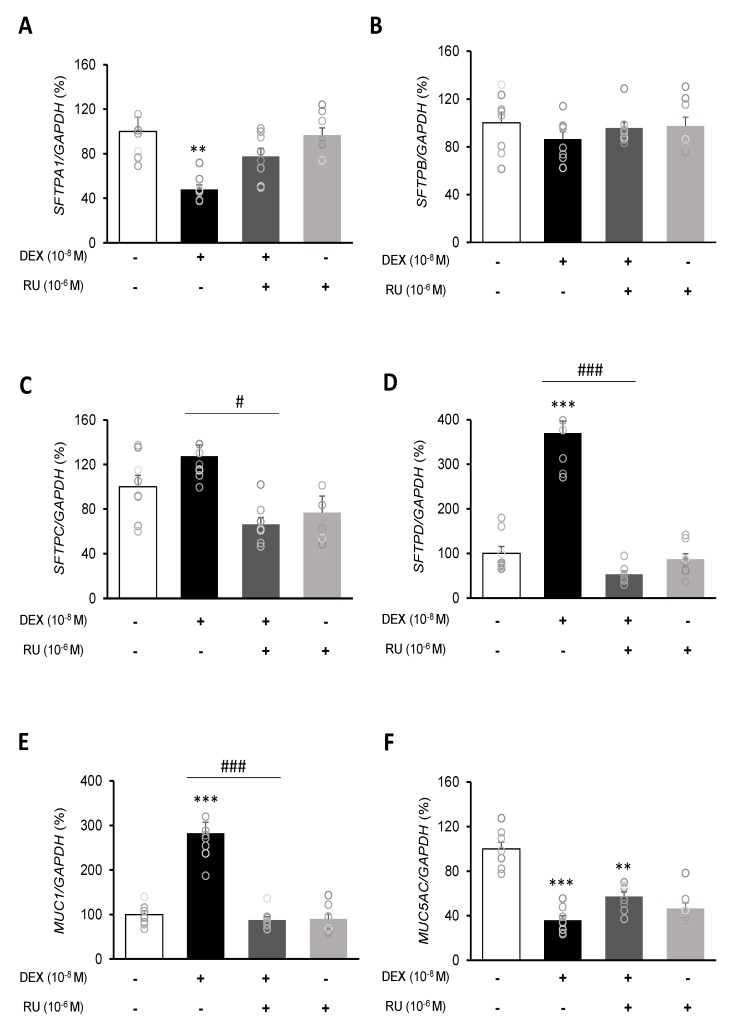
Regulation of surfactant protein and mucin genes expressions by dexamethasone in A549 cells. Effect of DEX and its antagonist (RU486) on the transcriptional level of (**A**) surfactant protein A (SFTPA1), (**B**) surfactant protein B (SFTPB), (**C**) surfactant protein C (SFTPC), (**D**) surfactant protein D (SFTPD), (**E**) mucin 1 (MUC1), and (**F**) mucin 5AC (MUC5AC) by real-time PCR. The mRNA level was measured by performing real-time PCR and was normalized by GAPDH. ** *p <* 0.01 versus Control; *** *p <* 0.001 versus control; ^#^ *p <* 0.05 versus DEX; ^###^ *p <* 0.001 versus DEX.

**Figure 6:**
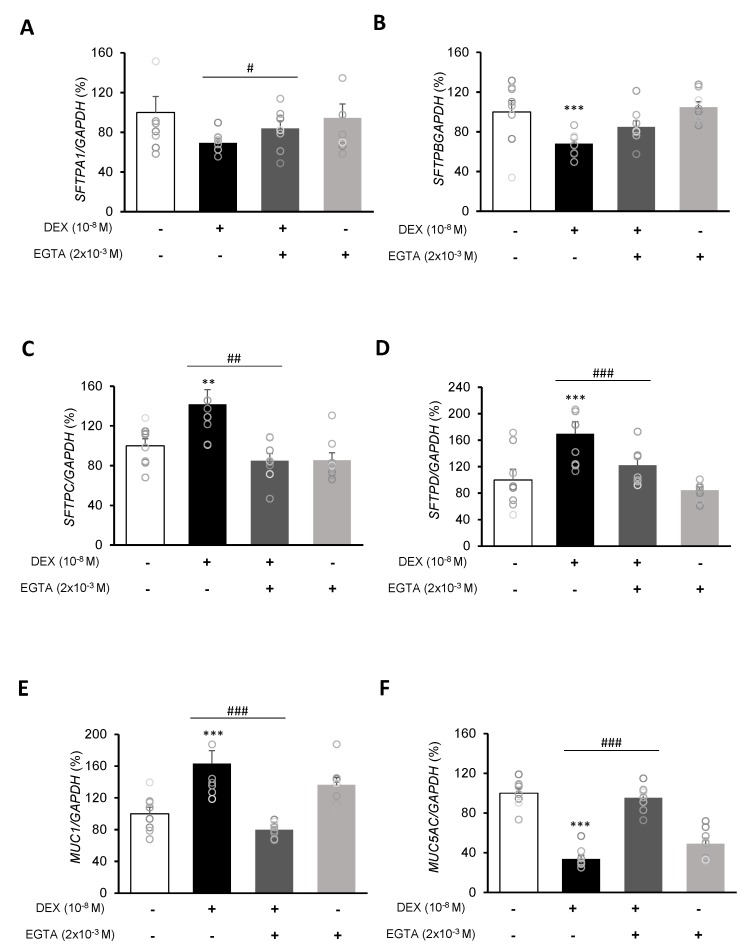
Effect of EGTA on surfactant protein and mucin genes in A549 cells. Effect of EGTA and DEX on the transcriptional level of (**A**) surfactant protein A (SFTPA), (**B**) surfactant protein B (SFTPB), (**C**) surfactant protein C (SFTPC), (**D**) surfactant protein D (SFTPD), (**E**) mucin 1 (MUC1), and (**F**) mucin 5AC (MUC5AC) by real-time PCR. The mRNA level was measured by performing real-time PCR and was normalized by GAPDH. ** *p <* 0.01 versus Control; *** *p <* 0.001 versus control; ^#^ *p <* 0.05 versus EGTA; ^##^ *p <* 0.01 versus EGTA; ^###^ *p <* 0.001 versus EGTA.

To investigate the mRNA expressions of pulmonary surfactant genes *SFTPA1*, *SFTPB*, *SFTPC*, and *SFTPD* and the secreted airway mucin genes *MUC1* and *MUC5AC*, DEX was treated to A549 cells for 24 h. DEX significantly decreased the mRNA expressions of *SFTPA1* and *SFTPB* (Figure 5A,B) but significantly increased those of *SFTPC* and *SFTPD* (Figure 5C,D). The DEX plus RU486 or EGTA treatments significantly increased the mRNA expressions of *SFTPA* and *SFTPB* (Figure 6A,B) and significantly decreased the mRNA levels of *SFTPC* and *SFTPD* (Figure 6C,D). The membrane-bound mucus *MUC1* mRNA level was significantly increased by DEX treatment compared to that in the control cells without DEX (Figure 5E), whereas *MUC5AC* mRNA was significantly decreased (Figure 5F). The DEX plus RU486 or EGTA treatments inversely recovered the mRNA levels of *MUC1* and *MUC5AC* (Figure 6E,F).

These results indicate that DEX influences the intracellular calcium level through regulation of the *TRPV6* and *NCX1* genes in A549 cells. In addition, DEX affects the pulmonary surfactant genes *SFTPA*, *SFTPB*, *SFTPC*, and *SFTPD* and the secreted airway mucin genes *MUC1* and *MUC5AC* in A549 cells.

## 3. Discussion

Calcium is an important physiological component in many cells. Several researchers have reported that calcium-processing genes can be regulated by steroid hormones [3,19]. However, the regulation mechanism of calcium-processing genes in lung cancer cells has not been fully decribed. In the present study, the effects of the corticosteroid DEX on intracellular calcium concentrations and on expressions of calcium-processing genes in the A549 cell line, a human type 2 alveolar lung cancer cell line, were investigated. Significant changes were shown in the expressions of calcium-processing and secretion-related genes in the DEX-treated group. Previous studies have shown that TRPV6, NCX1, and PMCA1 levels are regulated in many tissues by steroid hormones such as DEX [3,20]. DEX has been shown to increase TRPV6 and NCX1 expressions in the lungs of mice, but there was no DEX-induced change in NCX1 expression in mouse kidney [3,21].

DEX treatment can activate either large-conductance calcium-activated potassium channels or epithelial sodium channels (ENaCs), which may be present in A549 cells, via a non-genomic mechanism [22,23,24]. Since A549 cells appear to be not electrically excitable, an increase in the electrochemical driving force due to the activation of such channels could facilitate calcium influx from the exterior, with such an influx being independent of TRPV or NCX1 activity. Therefore, the possibility that the DEX-induced increase in the intracellular calcium level in alveolar epithelial A549 cells could be linked to its effects on the other channels, could not be excluded. In addition, TRPV or NCX1 gene expressions by RU486 could be partly and indirectly attributed to its suppression in calcium currents. Acute treatment with DEX or mifepristone (RU486) might suppress calcium channels in electrically excitable cells, such as motor neurons and vascular smooth muscle cells (VSMCs) [25,26]. Therefore, a DEX-mediated change in intracellular calcium level may be discernable in electrically excitable cells such as tracheal smooth muscle cells.

Figure 3 and Figure 5 show that the results of treatment with RU486 alone are similar to those of treatment with DEX plus RU486. RU486 has two different mechanisms that can regulate calcium concentration: the inhibition of calcium influx and stimulation of calcium efflux. In addition, RU486 stimulates calcium storage in some intracellular organelles [27,28]. Lobaccaro-Henri et al. [27] indicate that RU 486 could act as an inhibitor of intracellular calcium mobilization in human myometrium. Especially, Serres et al. [28] demonstrate that RU486 suppresses the increase in basal calcium influx by progesterone in spermatozoa. RU486 does not necessarily compete for the same binding sites as progesterone to exert its inhibitory action. In addition, RU486 acts at the membrane level of some intracellular organelles of calcium storage. In this study, expression of the calcium influx gene *TRPV6* was decreased by RU486-only and DEX plus RU486 treatments, whereas expression of the calcium efflux gene *NCX1* was increased by those treatments. In addition, RU486-only and DEX-plus RU486 treatments increased the mRNA expressions of *SFTPA1*, *MUC1*, *MUC5AC*, and decreased the mRNA levels of *SFTPC* and *SFTPD*.

The expression of TRPV6 in uterus and the placenta is regulated via progesterone-receptor- or estrogen-receptor-mediated pathways during pregnancy in rodents [29]. TRPV6 is expressed in placenta-unattached areas and luminal and glandular epithelial cells of the uterus, whereas TRPV6 is detected in the labyrinth and spongy zone of the placenta. In rats, TRPV6 expression was reduced by RU486 via progesterone receptors, while, in mice, ICI 182,780 (an anti-estrogen) blocked TRPV6 expression via estrogen receptors. The juxtaposition of uterine and placental TRPV6 expression supports the suggestion that TRPV6 participates in transferring calcium ions between maternal and fetal compartments.

To confirm that DEX can regulate intracellular calcium levels, we investigated intracellular calcium concentrations using the pGP-CMV-GCaMP6f, CMV-R-GECO1.2 calcium indicator and the Rhod-4 assay. GCaMP6f and GECO1.2 is a powerful indicator used in calcium imaging that enables the detection of calcium signals [15,16,30]. The Rhod-4 analysis is a fluorescence-based assay used in the detection of intracellular calcium mobilization [31]. In previous studies, both the pGP-CMv-GCaMP6f and Rhod-4 indicators are strongly expressed when the intracellular calcium concentration is increased [15,16]. Our results confirmed that DEX treatment can increase intracellular calcium concentration.

We investigated the relationship between intracellular calcium concentration and secretion-related gene expressions following DEX treatment. The quantities of surfactant proteins and mucins are important for the maintenance of lung function. Lung surfactant proteins are divided, depending on their properties, into the following: hydrophilic SFTPA1 and SFTPD and hydrophobic SFTPB and SFTPC [10]. The SFTPA1 and SFTPD proteins lower surface tension and serve as primary defenses against microbes [32,33]. The SFTPB and SFTPC proteins help to maintain the integrity of the alveoli by inserting phospholipids at the air–liquid interface [10,34]. Corticosteroids are reported to affect mucin expression [10,35]. Previous studies have demonstrated that the levels of pulmonary surfactants SFTPB, SFTPC, and SFTPD are significantly increased by DEX treatment in lung cell lines A549 and H441 [6]. Consistent with the results reported by Rucka et al. [6], the present study demonstrated that the expression of SFTPA was reduced by DEX treatment, while the expressions of SFTPC and SFTPD were significantly increased, but the change in SFRPB level was not significant. In addition, the expression of *MUC1* was increased and that of *MUC5AC* was decreased. The increase in *MUC1* expression is consistent with that observed in cancer cell lines [36] and the decrease of *MUC5AC* expression is in agreement with previous results in lung epithelial cells [12,37]. These results indicate that DEX regulates the expression of secretion-related genes. Changes in the expression of surfactant protein and mucin genes are observed in many lung diseases, such as chronic inflammatory lung disease and lung disorders, and the results of the present study provide a useful basis for further lung disease-related studies [38].

Based on our results, and after considering changes in the expression of calcium-processing genes and changes in surfactant protein and mucin genes, one hypothesis can be proposed: DEX treatment increases the calcium influx TRPV6 level while it decreases that of calcium release NCX1, as a result, intracellular calcium levels increase, inducing the expression of surfactant protein and mucin genes. An et al. [3] suggested that increased expressions of calcium-processing genes have an effect on the expressions of mucin-secreting genes, and our results are consistent with those results. In order to identify the relationship between changes in calcium-processing gene expressions and those in secretion-related genes, we examined changes in surfactant protein and mucin genes by treating A549 cells with the calcium-specific chelator EGTA. The EGTA treatment reversed the effect of DEX treatment; the expression of *TRPV6* was increased by EGTA and that of *NCX1* was decreased. In addition, the expressions of *SFTPA* and *SFTPB* were increased and those of *SFTPC* and *SFTPD* were significantly decreased. The expression of *MUC1* was decreased and that of *MUC5AC* was increased by EGTA. Therefore, changes in calcium levels under EGTA treatment may affect secretion-related genes.

The TRPV channels and NCX1 regulate mucin secretion in colon cells [39,40]. In addition, mucin expression is regulated by PMCA1 in the epithelial cells of the digestive tract [14]. Associations between calcium-processing genes and secretory genes have not been fully determined. Therefore, further studies on the relation between calcium-processing genes and secretory-related genes are needed for research on lung disease.

**Figure 7:**
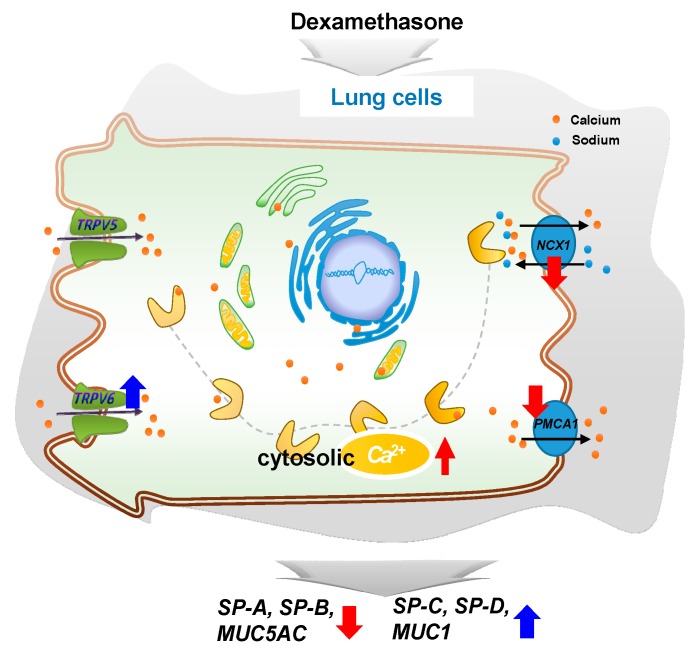
A simplified schematic diagram of the DEX effect on the intracellular calcium level through *TRPV6* in A549 cells. DEX influences the intracellular calcium level through *TRPV6* and affects the pulmonary surfactant genes (*SFTPA*, *-B, -C,* and *-D*) and the secreted airway mucin genes (*MUC1* and *MUC5AC*) in A549 cells. **↑**, increase; **↓**, decrease.

In conclusion, DEX regulates intracellular calcium levels through the regulation of *TRPV6*, *NCX1*, and *PMCA1* in A549 cells and affects the expressions of surfactant protein and mucin genes. This study confirms that steroid hormones in the lungs can regulate the function of the lungs by varying calcium levels. Our observations may provide a basis for further studies on the regulation of surfactant proteins and mucin genes (Figure 7).

## 4. Methods

### 4.1. Cell Culture and Treatments

The A549 cells are adenocarcinomic human alveolar basal epithelial cells and have served as models of human alveolar type 2 pulmonary epithelium in investigations into the metabolic processing of lung tissue and possible mechanisms of drug delivery to such tissue. The A549 cells were purchased from the Korean Cell Line Bank. The A549 cells were cultured in basal medium and grown in 100 mm plates (Corning Inc., Corning, NY, USA) at 37 °C in 5% CO_2_. The basal medium consisted of DMEM (Biowest, USA) supplemented with certified fetal bovine serum (FBS; Biowest Nuailléa, France), penicillin (100 U/mL) and streptomycin (100 µg/mL; Biowest). To confirm the effect of DEX in the A549 cell line, A549 cells were transferred to DMEM without phenol red and with 5% charcoal/dextran-treated FBS instead of normal FBS. Adherent cells were grown for 1 day, and, on the next day (2 day), the A549 cells were treated with 10^−8^ M DEX for 24 h. Some of the 10^−8^ M DEX-treated groups were also treated with 10^−6^ M of RU486, a DEX antagonist.

### 4.2. Intracellular Calcium Response

#### 4.2.1. Rhod-4 Calcium Response Assay 

Calcium imaging was performed using an automated microscope (BioTek Lionheart FX, Miami, FL, USA). The A549 cells were adhered to a cover glass bottom plate (SPL Life Science, Seoul, Republic of Korea) in shielded medium for 1 d and then treated with DEX for 24 h. The A549 cells were then reacted with 200 μL/well of a Rhod-4 dye-loading solution (Abcam, ab112157) at 37 °C for 30 min in a calcium-free buffer. Calcium imaging was performed at 3 s intervals for 20 min by using a confocal laser-scanning microscope. The A549 cells were treated with CaCl_2_ (2 mM) for 20 s to measure the calcium response in the cytosol.

#### 4.2.2. Calcium Response Assay with pGP-CMV-GCaMP6f and CMV-R-GECO1.2 Transfection

The A549 cells were seeded at 3 × 10^5^ cells/well and co-transfected with 0.5 μg of pGP-CMV-GCaMP6f (Addgene, MA, USA) and CMV-R-GECO1.2 (Addgene, MA, USA) 1.5 μL of Lipofectamine (Invitrogen, Lipofectamine 2000, Carlsbad, USA) in 50 μL of Opti-MEM medium (Gibco, USA) at room temperature for 5 min. The DNA–lipid complex was mixed with the cells and the mixture incubated at 37 °C for 1 day. After confirming the presence of transfected cells, DEX was treated for 6, 12, and 24 h prior to determine calcium concentration by using an automated microscope (BioTek, Lionheart FX).

### 4.3. Western Blot Analysis

The A549 cells were lysed using RIPA buffer (Invitrogen, USA). The BCA assay (Sigma-Aldrich) was performed at a 562 nm absorbance to determine protein concentration. Protein (40 μg) was analyzed by 7.5% SDS-PAGE and then electrophoresed onto a PVDF membrane. The PVDF membrane was incubated with primary antibodies for 1 d. Primary antibodies to β-actin (1:1000, 4970S, Cell Signaling Technology, Danvers, MA, USA), TRPV6 (1:1000, ACC-036, Alomone Labs, Jerusalem, Israel), NCX1 (1:500, MA3-926, Thermo Fisher Scientific, MA, USA), and PMCA1 (1:1000, Swant, Marly, Switzerland) were used. After incubation with primary antibodies, membranes were treated with secondary antibodies (anti-rabbit, 1:3000, Cell Signaling Technology; anti-mouse, 1:3000, Cell Signaling Technology) for 1 h at room temperature. The membrane was then washed with 1× TBS-T three times each for 10 min at room temperature. An ECL solution using Chemi Doc, GenGnome 5 (Syngene, Cambridge, UK) was used to confirm antibody binding. Image J v1.37 software (Wayne Rasband, NIH, Bethesda, MD, USA) was used for target band analysis.

### 4.4. Preparation of RNA and Real-Time PCR

The A549 cells were washed with PBS and treated with TRIzol (Ambion, Austin, TX, USA). The total RNA concentration was determined at an absorbance of 260 nm. RNA (1 µg) was transcribed by using MMLV (iNtRON Bio, Gyeonggi-do, Republic of Korea) with a 9 mer random primer (TaKaRa Bio, Kusatsu, Japan) to produce complementary DNA (cDNA).

The 2 µL cDNA template was analyzed by using SYBR Premix Ex Taq (TaKaRa Bio). Real-time PCR was performed with 10 pM of each specific primer. The primer sequences are listed in Table 1. The real-time PCR was performed in 40 cycles. The cycle conditions were denatured at 95 °C for 30 sec, annealing at 58 °C for 30 sec, and extended at 72 °C for 30 s using a QuantStudio 3 Real-Time PCR system (Applied ThermoFisher, USA). GAPDH was used for the internal control and the relative gene expression levels were calculated using the 2^Δ*C*T^ method.

### 4.5. Data Analysis

The data are presented as mean ± SEM values of three or more experiments and were analyzed by using one-way ANOVA. Statistical analysis was performed using Prism Graph Pad software (v5.0; GraphPad Software Inc., San Diego, CA, USA).

## Figures and Tables

**Table 1:** Oligonucleotide sequences for quantitative real-time PCR.

Gene	Primer Sequence (5′→3′)	Accession Number
*TRPV6*	F: GGAGCTAGGCCACTTCTACG	NM_018646.6
	R: ATGGCAATGAGGAGGTTGAG	
*NCX1*	F: GACCTCGGTCCTAGCACCAT	NM_001112800.2
	R: ACACCAGGAGATATGACAGACAA	
*PMCA1*	F: GCTGGAGGTGAAGAGGAA	NM_001001323.2
	R: GCACTGCGACCACTAAAA	
*SFTPA1*	F: TTTGATGCCATTCAGGAGGC	NM_001093770.3
	R: TCGGTACCAGTTGGTGTAGT	
*SFTPB*	F: AAGTTCCTGGAGCAGGAGTG	NM_000542.5
	R: AGAGGAATGGGGAATTGCTG	
*SFTPC*	F: GGCACCTGCTGCTACATCAT	NM_001172357.2
	R: CCAGCTTAGACGTAGGCACT	
*SFTPD*	F: CTTCTCTGAGGCAGCAGGTT	NM_003019.5
	R: CTGTGCCTCCGTAAATGGTT	
*MUC1*	F: ATCTCATTGCCTTGGCTGTC	NM_001018016.3
	R: TAGGGGCTACGATCGGTACT	
*MUC5AC*	F: AACCAGTCGACCTGTGCTGT	NM_001304359.2
	R: TCGAGCGAGTACATGGAAGA	
*GAPDH*	F: AAGGTCATCCCTGAGCTGAA	NM_001256799.3
	R: GGGAGCCAAAAGGGTCATCA	

F: forward; R: reverse.
